# Random Subspace Aggregation for Cancer Prediction with Gene Expression Profiles

**DOI:** 10.1155/2016/4596326

**Published:** 2016-11-24

**Authors:** Liying Yang, Zhimin Liu, Xiguo Yuan, Jianhua Wei, Junying Zhang

**Affiliations:** ^1^School of Computer Science and Technology, Xidian University, Xi'an, Shaanxi 710071, China; ^2^State Key Laboratory of Military Stomatology, Department of Maxillofacial Surgery, School of Stomatology, the Fourth Military Medical University, Xi'an, China

## Abstract

*Background*. Precisely predicting cancer is crucial for cancer treatment. Gene expression profiles make it possible to analyze patterns between genes and cancers on the genome-wide scale. Gene expression data analysis, however, is confronted with enormous challenges for its characteristics, such as high dimensionality, small sample size, and low Signal-to-Noise Ratio.* Results*. This paper proposes a method, termed RS_SVM, to predict gene expression profiles via aggregating SVM trained on random subspaces. After choosing gene features through statistical analysis, RS_SVM randomly selects feature subsets to yield random subspaces and training SVM classifiers accordingly and then aggregates SVM classifiers to capture the advantage of ensemble learning. Experiments on eight real gene expression datasets are performed to validate the RS_SVM method. Experimental results show that RS_SVM achieved better classification accuracy and generalization performance in contrast with single SVM, *K*-nearest neighbor, decision tree, Bagging, AdaBoost, and the state-of-the-art methods. Experiments also explored the effect of subspace size on prediction performance.* Conclusions*. The proposed RS_SVM method yielded superior performance in analyzing gene expression profiles, which demonstrates that RS_SVM provides a good channel for such biological data.

## 1. Introduction

Cancer usually has an association with genes which carry human heritage information. Completion of human genome sequencing makes genetic analysis on the genome-wide scale available and provides a deeper understanding of the underlying mechanism of cancers [1–4]. Biological technology now can simultaneously monitor ten thousands of gene expression levels [5, 6]. It is meaningful to design novel methods to precisely and efficiently classify tumor samples from normal samples or recognize subclasses of some disease with gene expression profiles. Classification of gene expression data, however, faces enormous difficulties. Firstly, the data have up to ten thousands of dimensions. Traditional classification methods become intractable, since high dimensionality makes sample distribution dispersing and distance between samples ambiguous. Secondly, sample size is small for high expenses or ethical consideration. Therefore, there is no enough data to train a classical learner. Low Signal-to-Noise Ratio (SNR) is the third issue to consider for gene expression data analysis, which means noise may significantly decline performance.

To tackle the high dimensionality issue, some researches make an attempt to select important gene features by exploiting the association among genes and eliminating redundant and irrelevant information. Based on Recursive Feature Elimination (RFE), Guyon et al. used SVM method to select genes and proved that the genes filtered by SVM method perform better [7]. By feature extraction and defining “correlation feature space” for samples built on gene expression profiles through iterative utilization of Pearson's correlation coefficient, Ren et al. proposed an original method to further propel gene expression profiling technologies from bench to bedside [8]. Considering the possible interactions among genes, Zhang et al. proposed a binary matrix shuffling filter to surmount troubles linked with searching schemes of conventional wrapper method and overfitting [9].

Ensemble art is also introduced in some recent researches. Bolón-Canedo et al. provided a novel framework for feature selection by an ensemble of filters and classifiers [10]. Combining classifiers from different classification families into an ensemble based on the evaluation of performance of each classifier, Nagi and Bhattacharyya proposed an ensemble method named as SD-EnClass [11]. To ensure a high classification accuracy, Ghorai et al. showed an ensemble of nonparallel plane proximal classifiers based on the genetic algorithm through simultaneous feature and model selection scheme [12]. Given the fact that forward feature selection (FFS) method is able to obtain an expected feature subset with less iteration than that of backward feature selection (BFS) method, Luo et al. proposed two FFS methods based on the pruning of the classifier ensembles generated by a single gene feature [13].

“Blessing of nonuniformity” effect, which means samples are concentrated in a relatively low instance space rather than uniformly throughout the whole space, inspired some novel methods to perform classification in subspaces [14]. Constructing subspace in random process was firstly proposed by Ho for decision forests to overcome the dilemma between avoiding overfitting and achieving maximum accuracy [15].

Recently, researchers have done much work on cancer classification based on gene expression data. Daxa et al. proposed a framework to find informative gene combinations and to classify gene combinations belonging to their relevant subtype by using fuzzy logic, while they only focused on identifying 2-gene and 3-gene combinations [16]. Kim et al. presented a genetic filter to identify gene subset for cancer-type classification on gene expression profiles, which was only tested on one dataset, that is, Leukemia dataset [17]. Vosooghifard and Ebrahimpour proposed a hybrid method using GWO and C4.5 for gene selection and cancer classification. In essence, GWO is a group optimization method, so time consuming is a factor which should be considered [18]. Buza summarized the classification of gene expression data in reference [19], where he indicated that the robustness of SVM to classify gene expression data relies on the strong fundamentals of statistical learning theory.

This paper attempts to classify gene expression data by aggregating SVMs trained on random subspaces (RS). RS method shows great potential in scenarios where the number of features is much bigger than the number of samples [20–23]. In addition, RS method has an excellent performance in coping with correlation and redundancy between features. Bias risk is relatively small in RS because of its independence of specific hypothesis on data. SVM is usually used to cope with gene expression data, since only support vectors work in classification process, and the number of support vectors is usually much smaller than that of training samples. We elaborately explored the trick of choosing parameters and the effect of size of subspaces on the classification performance. The possible reason leading to unsatisfied outcome was also revealed.

## 2. Materials and Methods

### 2.1. Gene Expression Datasets

Eight real gene expression datasets are used. They are provided by Kent Ridge Biomedical Dataset Repository and collected by Li and Liu from Nanyang Technological University, Singapore [24]. Detailed information is listed in Table 1.

Breast Cancer dataset labels the patients who had got distance metastases in five years as “relapse” and label the patients who remained healthy since the initial diagnosis for interval of at least five years as “nonrelapse.” Missing values are replaced by 100 [25].

Leukemia dataset was originally published in reference [26]. Dataset used in this work is an extended and more heterogeneous version than the initial one. Samples are from DFCI (Dana-Farber Cancer Institute), CALGB (Cancer and Leukemia Group B), and SJCRH (St. Jude Children's Research Hospital). There are two categories, ALL (Acute Lymphoblastic Leukemia) and AML (Acute Myeloid Leukemia), inside the total 72 samples over 7129 probes. Training dataset consists of 38 bone marrow samples (27 ALL and 11 AML), while 34 testing samples (20 ALL versus 14 AML) are provided with 24 bone marrow and 10 peripheral blood specimens.

Lung Cancer dataset was firstly presented in reference [27]. Training set consists of 16 malignant pleural mesothelioma (MPM) samples and 16 adenocarcinoma (ADCA) samples. Testing set contains 15 MPM samples and 134 ADCA samples. 12533 genes expression levels were obtained via hybridizing cRNA to human U95A oligonucleotide probe arrays. All the ADCA samples and 12 MPM samples were processed at the Dana-Farber Cancer Institute and the Whitehead Institute. The remaining 19 MPM samples were processed separately at Brigham and Women's Hospital.

Prostate dataset has an independent testing set, which is from a different experiment and has a nearly tenfold difference in overall microarray intensity from the training data [40].

Colon Tumor dataset was introduced in reference [41]. Rather than elaborating time-course data, this dataset consists of snapshots of the expression pattern of distinct cell types. Raw dataset, based on 22 normal colon tissue samples (positive) and 40 colon tumor samples (negative) of colon adenocarcinoma specimens, was from an Affymetrix oligonucleotide array complementary to more than 6,500 genes and expressed sequence tags (ESTs). Two thousand genes were selected to generate the dataset used here, with the highest minimal intensity across 62 samples.

CNS (central nervous system) dataset was originally published in reference [42], while only dataset C mentioned to analyze the outcome of the treatment is used here. 60 samples consist of 39 medulloblastoma survivors (Class 0) and 21 treatment failures (Class 1). The dataset contains 60 patient samples, with 21 medulloblastoma survivors (labelled as “Class 1”) and 39 treatment failures (labelled as “Class 0”). There are 7129 genes in the dataset.

Ovarian dataset was originally published in reference [43], inside which experiments are to identify proteomic patterns in serum that distinguish ovarian cancer from noncancer. The proteomic spectra were generated by mass spectroscopy and dataset used in this work includes 91 “Normal” samples and 162 “Cancer” samples without separated training set and testing set. The raw spectral data of each sample contains the relative amplitude of the intensity at each molecular mass/charge (*M*/*Z*) identity. There are totally 15154 *M*/*Z* identities. The intensity values were normalized according to the formula NV = (*V* − Min)/(Max − Min), where NV is the normalized value, *V* the raw value, Min the minimum intensity, and Max the maximum intensity. The normalization is done over all the 253 samples for all 15154 *M*/*Z* identities. Thus, each intensity value falls into the range of 0 to 1.

As the most common subtype of non-Hodgkin's lymphoma, DLBCL (diffuse large B cell lymphoma) is due to an aggressive malignancy of mature B lymphocytes. DLBCL consists of two molecularly different subclasses [44]. One subclass is “germinal centre B like DLBCL” expressing gene characteristics of germinal centre B cells and the other is “activated B-like DLBCL” expressing genes normally induced during* in vitro* activation of peripheral blood B cells. DLBCL dataset contains 47 mRNA samples consisting of 24 germinal centre B-like DLBCL and 23 activated B-like DLBCL. Each of 4026 column score responding to cDNA clones indicates a gene expression level. Log-transformation was implemented on raw dataset to produce the dataset used in this work.

### 2.2. Method Description

**Figure 1:**
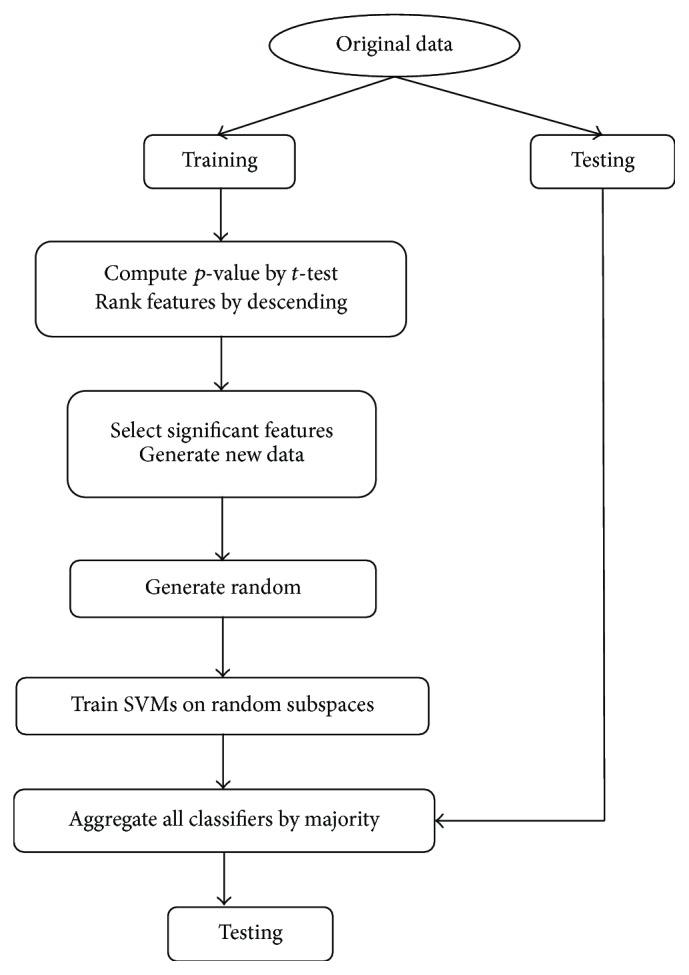
RS_SVM method.

SVM has an advantage in small sample cases and RS method shows an excellent performance in coping with high-dimension data.  Algorithm 1 presents a description of RS_SVM method used in this paper, which aggregates SVMs trained on random subspaces.  Figure 1 shows the framework of RS_SVM.

### 2.3. Gene Selection

Gene expression profile usually contains a large number of genes with constant or near constant expression levels across samples. These genes are redundant for classification and even decline distinction between normal and tumor samples, since they sharply increase space dimensions. To address this problem, gene selection based on statistical analysis is adopted to yield a new gene set from the original one. Since *t*-test is the first method for feature selection when microarray technology came into being, it is used in this work. Firstly, we compute *p* value of each gene across total samples and rank genes according to *p* value; then, top genes are filtered at 0.95 significant level. Number of top genes and optimal size of subspace on eight datasets are presented in Table 2.

### 2.4. Size and Number of Random Subspaces

Random subspace size (*S*) has an enormous influence on RS_SVM. Supposing that *S* value is relatively small, some important gene features may not be selected into feature subsets to train SVMs; thus, underfitting easily occurs. In contrast, if *S* is extremely large, diversity among SVM classifiers may be reduced, leading to a useless aggregation. Following experiment sets, default *S* to be the square root of *M* (feature number of selected data by *t*-test), recommended by Breiman [45], and then adjust *S* until achieving the optimal testing error. We analyze the influence of random subspace size on classification performance via illustrating the variation of training error and testing error with different *S* in Figure 3. An appropriate number of random subspaces (*L*) can guarantee that each feature has enough chance to be selected. Since the lack of prior knowledge about *L*, it is set to 1000 experimentally.

## 3. Results and Discussion

To validate the effectiveness of RS_SVM, we perform experiments on eight real gene expression datasets mentioned above. Three experiments are designed to validate the proposed method. In the first experiment, we computed testing error of RS_SVM and peer methods, including single SVM, KNN (*K*-nearest neighbor), CART (classification and regression tree), Bagging, and AdaBoost on eight datasets. Comparison of RS_SVM with the state-of-the-art methods in related literatures is also given. The second experiment explored influence of subspaces size by presenting the fluctuation of training error and testing error. In addition, sensitivity and specificity are also obtained at different subspace size. The last experiment shows the effectiveness of gene selection based on *t*-test.

The code is written in R-2.15.2, and all the packages are downloaded from the official site (https://www.r-project.org/).  Table 3 gives a detailed description of the functions, the relative parameters, and packages used in experiments. Note that there is no training set and testing set partition on Colon Tumor, CNS, Ovarian, and DLBCL; we perform leave-one-out cross validation on these datasets.

### 3.1. Testing Error Comparison of RS_SVM and Other Methods

Table 4 shows testing error of RS_SVM and other peer methods on eight datasets. Testing error of each method is computed on the same dataset. To eschew the interference of randomness, values in Table 4 are the average of 50 iterations. It is clear that RS_SVM performs best on five datasets, that is, Breast Cancer, Lung Cancer, Prostate, Ovarian, and DLBCL. It also achieves good results on Leukemia dataset. Effect of aggregation is obvious by comparing RS_SVM with single SVM, since testing error of RS_SVM is lower on six datasets, and RS_SVM obtains the same result with single SVM on Colon Tumor. The only exception is CNS. For CNS, all the methods do not perform well, which probably was due to the special distribution of data.

Table 5 shows testing error of RS_SVM and the state-of-the-art methods in literatures. It is obvious that none of these methods is always the winner, since distribution or correlation between gene features is diverse among different datasets. Each method has peculiar perspective for certain gene pattern. RS_SVM achieved the lowest testing error on Breast Cancer and Prostate and also relatively low testing error on the datasets of Leukemia, Lung Cancer, Ovarian, and DLBCL, which implies a good generalization performance.

**Figure 2:**
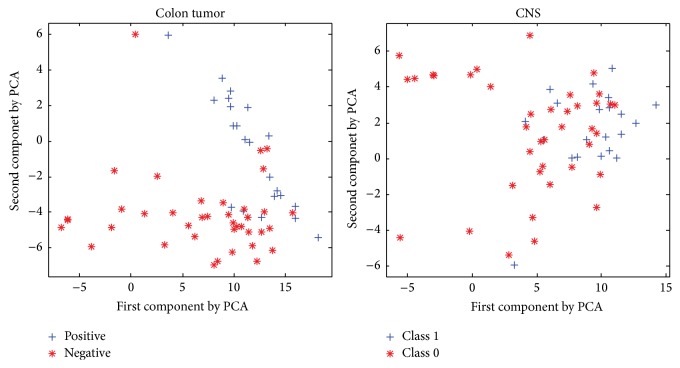
Scattering Colon Tumor and CNS data by Principle Component Analysis.

In spite of good performances mentioned in Tables 4 and 5, an unsatisfied outcome is revealed on Colon Tumor and CNS. Possible reason might be traced to heterogeneity phenomenon appearing in the two datasets [37], which means greater variability existing in gene expression level between the categories. To visually describe the distribution, Figure 2 projects high-dimension data to two-dimension space by Principle Component Analysis (PCA). Heterogeneity phenomenon is obvious in Colon Tumor and CNS data. For CNS, distribution of “Class 1” is relatively concentrated and “Class 0” is more dispersing. Similar case happens on Colon Tumor. This suggests that RS_SVM is not suitable for heterogeneous data.

### 3.2. Influence of Subspace Size

**Figure 3:**
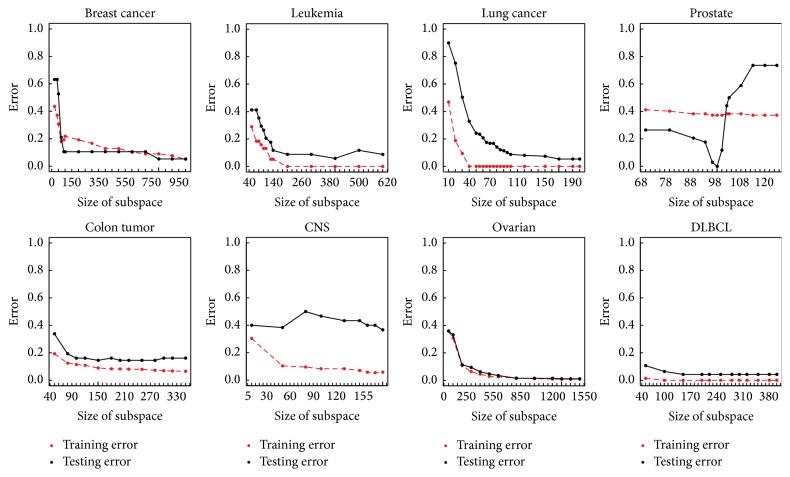
Variation of train error and test error with subspace size.

Figure 3 shows training error and testing error with respect to subspace size. Breast Cancer, Leukemia, Lung Cancer, Ovarian, and DLBCL share nearly similar curve trend. Initially, both training error and testing error are high when subspace size is small, which indicates underfitting exists. With the increasing of subspace size, both errors converge to nearly zero and underfitting fades away. However, the convergence rate is different among different datasets. Ovarian data converges much slower than the other four datasets. Errors of Ovarian are not near zero until subspace size is almost 800.

For Colon Tumor, when training error is near zero, there is a small gap between training and testing errors. This indicates that slight overfitting exists. More severe overfitting exists on CNS, because there is an obviously large gap between training error and testing error when training error is converging to zero. The terrible overfitting may explain RS_SVM's high testing error in Tables 4 and 5.

**Figure 4:**
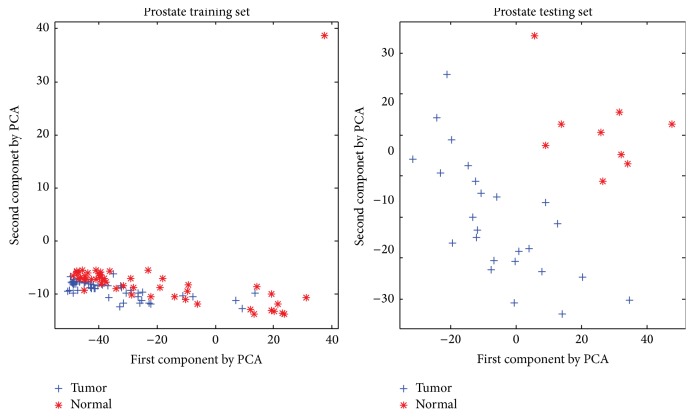
Scatter of training set and test set on Prostate based on the top two principle components.

For Prostate datasets, there is little variation on training error by increasing subspace size. Testing error, however, fluctuates dramatically, especially changing subspace size from 90 to 116. During this interval, testing error firstly drops down and minimum is obtained at the point when subspace size is set to 100, followed by rising up sharply, and finally tends to be steady. This phenomenon may be due to great differences between the distribution of training and testing set. As shown in Figure 4, tumor samples mainly concentrate in the left bottom in training set, while dispersing in the left in testing set. This indicates that the model generated on training set may not fit testing set well.

**Figure 5:**
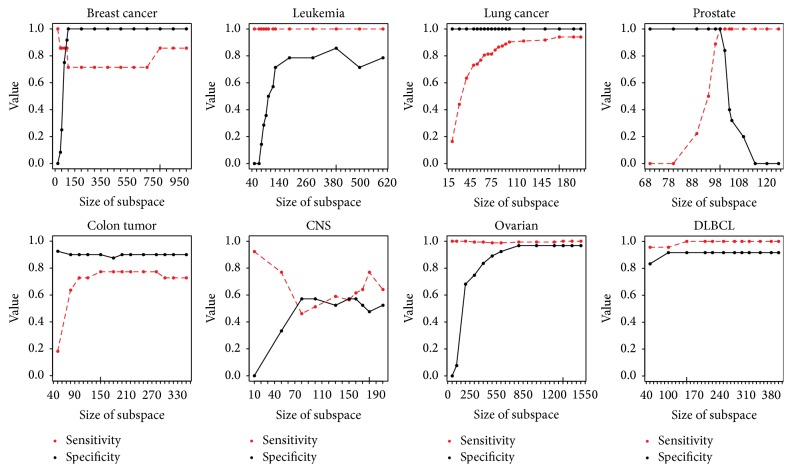
Variation of sensitivity and specificity with subspace size.

Figure 5 presents sensitivity and specificity with respect to subspace size. Sensitivity shows the ability to detect positives while specificity is the ability to reject negatives. To some extent, there is a trade-off between sensitivity and specificity. The best subspace size is a compromising value between sensitivity and specificity. For Breast Cancer, Leukemia, Lung Cancer, Ovarian, and DLBC, both sensitivity and specificity are high, which coincides with the low testing errors in Tables 4 and 5. Even though two curves of Colon Tumor are relatively steady, the whole level is not high. CNS dataset cannot achieve both high sensitivity and specificity, since when one rises up, the other drops down. The characteristic of Prostate dataset is also reflected in Figure 5. The sensitivity curve of Prostate rises up rapidly and then remains steady, but specificity curve drops down sharply when subspace size passes over the optimal value, which indicates that, with the increasing of subspace size, more and more tumor samples are predicted falsely.

### 3.3. Validation of Gene Selection by *t*-Test

The above experiments are performed on the datasets after gene selection via *t*-test, which is designed to reduce dimensionality and eliminate noise. In order to validate the effect of gene selection, we carry out experiment on datasets both with and without gene selection.  Table 6 gives the testing error of RS_SVM on eight datasets. For the sake of contrast, parameters of two cases are all uniform. Size of subspace chooses the optimal value obtained in Table 2. It shows that gene selection improves classification performance obviously by reducing testing errors.

## 4. Conclusions

This work proposed a cancer classification method, termed RS_SVM, to analyze gene expression profiles. The robustness of SVM relies on the strong fundamentals of statistical learning theory and the technique can be extended to nonlinear discrimination by embedding the data in a nonlinear space using kernel functions. In pattern recognition systems, no single model exists for all pattern recognition problems and no single technique is applicable to all problems. Ensemble learning is to integrate several models for the same problem. Random subspace is one of the ensemble learning methods and suitable for high-dimension data. For high-dimension gene expression data, only a small fraction of all genes is effective in performing certain diagnostic test. Hence, gene expression data analysis is confronted with enormous challenges for its characteristics, such as high dimensionality, small sample size, and low Signal-to-Noise Ratio. RS_SVM takes advantage of both subspace and SVM to handle the high-dimension and small sample problem in gene expression data, after obtaining the significant features through *t*-test, which could be regarded as prior knowledge to reduce the computing pressure. Experimental results on eight real gene expression profiles show that RS_SVM outperforms single SVM, KNN, CART, Bagging, AdaBoost, and 16 state-of-the-art methods in literatures. We also applied PCA on two gene expression profiles, where the experimental results are not satisfied, to probe the unsuitability. It suggests that RS_SVM is not suitable for heterogeneous data.

In RS_SVM, optimal values of subspace size and subspace number were obtained empirically, which was arduous and time-consuming. How to address this problem is still an open issue. We have collected next-generation sequencing gene expression data from TCGA and will continue this research on the new data.

## Figures and Tables

**Algorithm 1:**
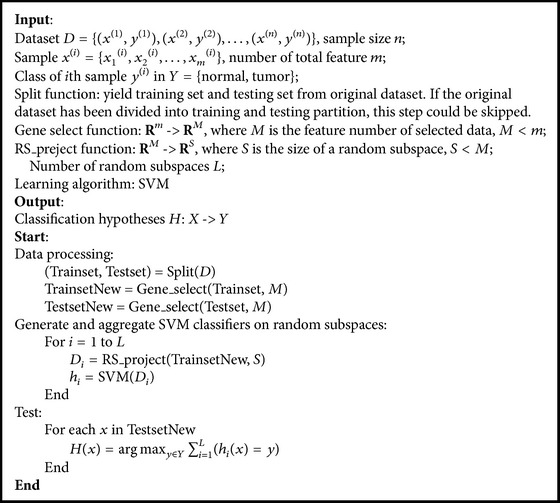


**Table 1:** Dataset.

Data	Feature	Sample	Class
Breast Cancer	24481	97 78 training (34 relapse + 44 nonrelapse) 19 test (12 relapse + 7 nonrelapse)	Relapse Nonrelapse

Leukemia	7129	72 38 training (27 ALL + 11 AML) 34 test (20 ALL + 14 AML)	All AML

Lung Cancer	12533	181 32 training (16 mesothelioma + 16 ADCA) 149 test (15 mesothelioma + 134 ADCA)	Mesothelioma ADCA

Prostate	12600	136 102 training (52 tumor + 50 normal) 34 test (25 tumor + 9 normal)	Tumor Normal

Colon Tumor	2000	62 22 positive + 40 negative	Positive Negative

CNS	7129	60 21 Class 1 + 39 Class 0	Class 1 Class 0

Ovarian	15154	253 162 cancer + 91 normal	Cancer Normal

DLBCL	4026	47 24 germinal + 23 activated	Germinal Activated

**Table 2:** Number of selected features and optimal size of subspace.

Data	Number of selected features by *t*-test	Optimal size of subspace
Breast Cancer	1810	800
Leukemia	1697	400
Lung Cancer	3134	170
Prostate	5707	100
Colon Tumor	394	150
CNS	378	180
Ovarian	7949	1300
DLBCL	972	150

**Table 3:** Function and package used in R.

Function	Package	Parameter
*t*.test()	stats	Confidence level of the interval is 0.95. Assume two variances are equal

svm()	e1071	Choose “radial” kernel; gamma is 1/dimension; epsilon is 0.1

knn()	class	Choose *k* = 3

rpart()	rpart	Choose method = “class”

ada()	ada	Use decision trees as base classifiers; iteration is 50; under exponential loss, type of boosting algorithm to perform is “discrete”

ipredbagg()	ipred	Use decision trees as base classifiers; number of bootstrap replications is 25

**Table 4:** Testing error comparison of RS_SVM and peer methods (%).

	RS_SVM	Single SVM	KNN	CART	AdaBoost	Bagging
Breast Cancer	**5.30**	15.79	47.37	31.58	10.53	31.58
Leukemia	5.89	26.47	**2.94**	8.82	41.18	8.82
Lung Cancer	**1.34**	9.40	2.68	9.40	51.01	9.40
Prostate	**0**	73.53	73.53	73.53	73.53	14.71
Colon Tumor	14.52	14.52	16.13	22.58	19.35	**11.29**
CNS	33.33	**31.67**	35.00	36.67	41.67	45.00
Ovarian	**1.19**	1.58	4.35	3.16	6.72	1.98
DLBCL	**4.26**	10.64	14.89	29.79	19.15	23.40

**Table 5:** Testing error comparison of RS_SVM and the state-of-the-art methods (%).

	Breast Cancer	Leukemia	Lung Cancer	Prostate	Colon Tumor	CNS	Ovarian	DLBCL
RS_SVM	**5.30**	5.89	1.34	**0**	14.52	33.33	1.19	4.26

Nanni et al. [28]	11.43	**0**	**0**	3.85	26.67	33.33	**0**	1.43

Ye et al. [29]	—	2.50	—	7.5	15.00	—	—	—

Liu et al. [30]	—	**0**	**0**	3.00	8.10	—	0.80	2

Tan and Gilbert [31]	—	8.90	6.80	26.50	4.90	11.7	—	—

Ding and Peng [32]	—	**0**	2.70	—	6.50	—	—	—

Bonilla Huerta et al. [33]	—	**0**	0.70	4.00	8.1	13.40	**0**	**0**

Cheng [34]	—	**0**	0.67	5.88	—	—	—	—

Paliwal and Sharma [35]	26.3	**0**	2.70	23.5	—	—	—	—

Bolón-Canedo et al. [10]	36.22	11.96	2.75	11.81	13.10	36.67	1.20	20.50
	46.56	4.11	**0**	41.87	16.19	30.00	0.8	6.50
	28.11	5.54	1.11	12.53	19.05	36.67	**0**	4.00

Porto-Díaz et al. [36]	21.05	**0**	0.67	20.59	10.00	25.00	**0**	**0**

Hu et al. [37]	—	—	12.50	19.30	9.70	—	—	—
	—	—	11.60	18.20	9.70	—	—	—

Nagi and Bhattacharyya [11]	26.51	7.55	18.12	47.06	5.60	**9.85**	1.11	

Pati and Das [38]	—	7.89	6.25	—	—	—	—	—

Dash et al. [39]	—	**0**	11.55	—	10.95	—	—	—
	—	0.45	**0**	—	**0**	—	—	—
	—	28.22	16	—	23.33	—	—	—
	—	0.41	0.95	—	0.31	—	—	—

Ghorai et al. [12]	18.79	5.48	3.62	9.84	17.23	—	—	—

Luo et al. [13]	—	2.07	—	—	18.60	—	—	6.00
	—	2.45	—	—	19.12	—	—	7.19

The state-of-the-art methods are indexed by the first author in literatures. “—” means that there are no corresponding results in the given literature.

**Table 6:** Effect of gene selection based on *t*-test (%).

	Breast Cancer	Leukemia	Lung Cancer	Prostate	Colon Tumor	CNS	Ovarian	DLBCL
With selection	**5.30**	**5.89**	**1.34**	**0**	**14.52**	**33.33**	**1.19**	**4.26**
Without selection	63.16	41.18	3.36	26.47	35.48	35.00	3.20	44.68
